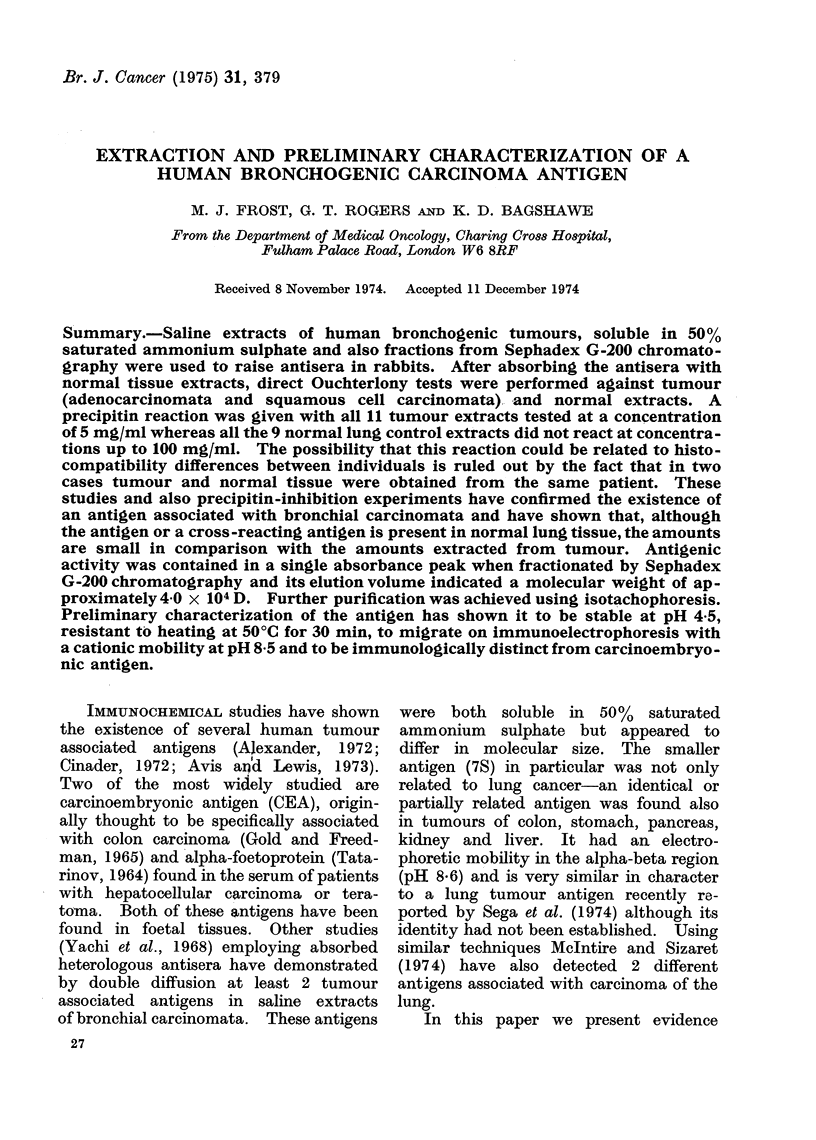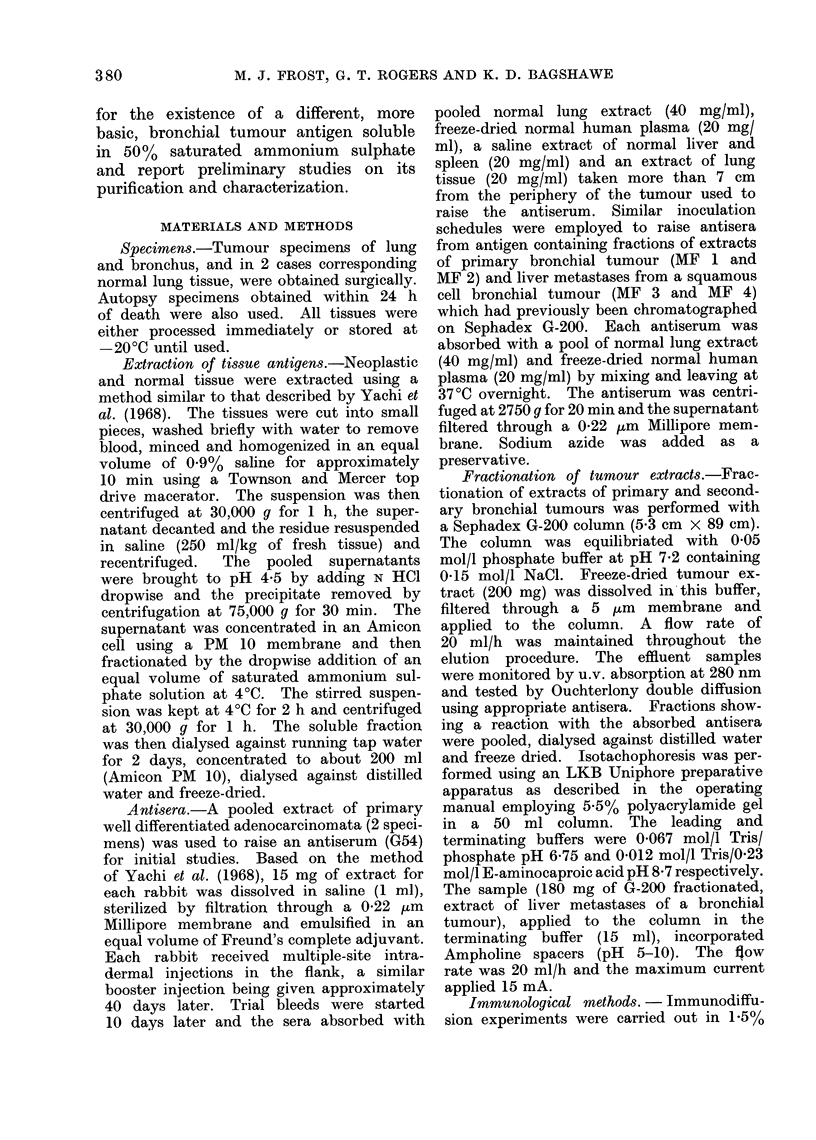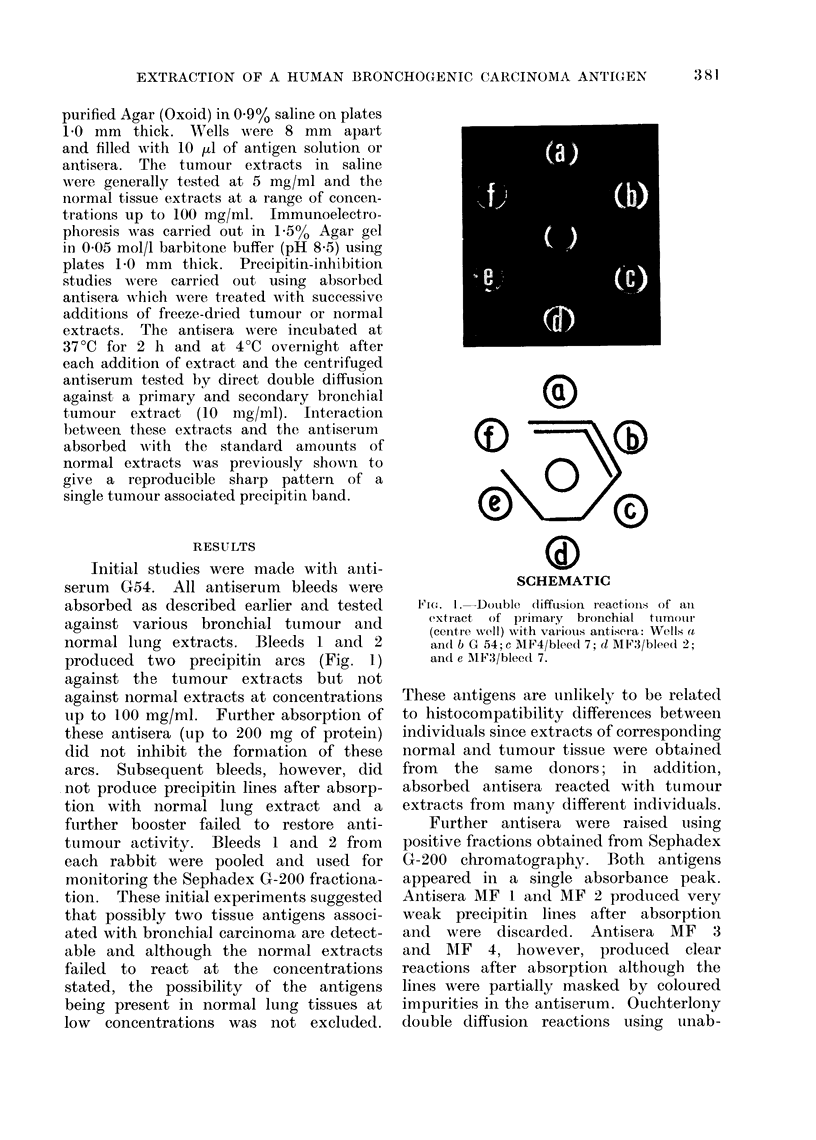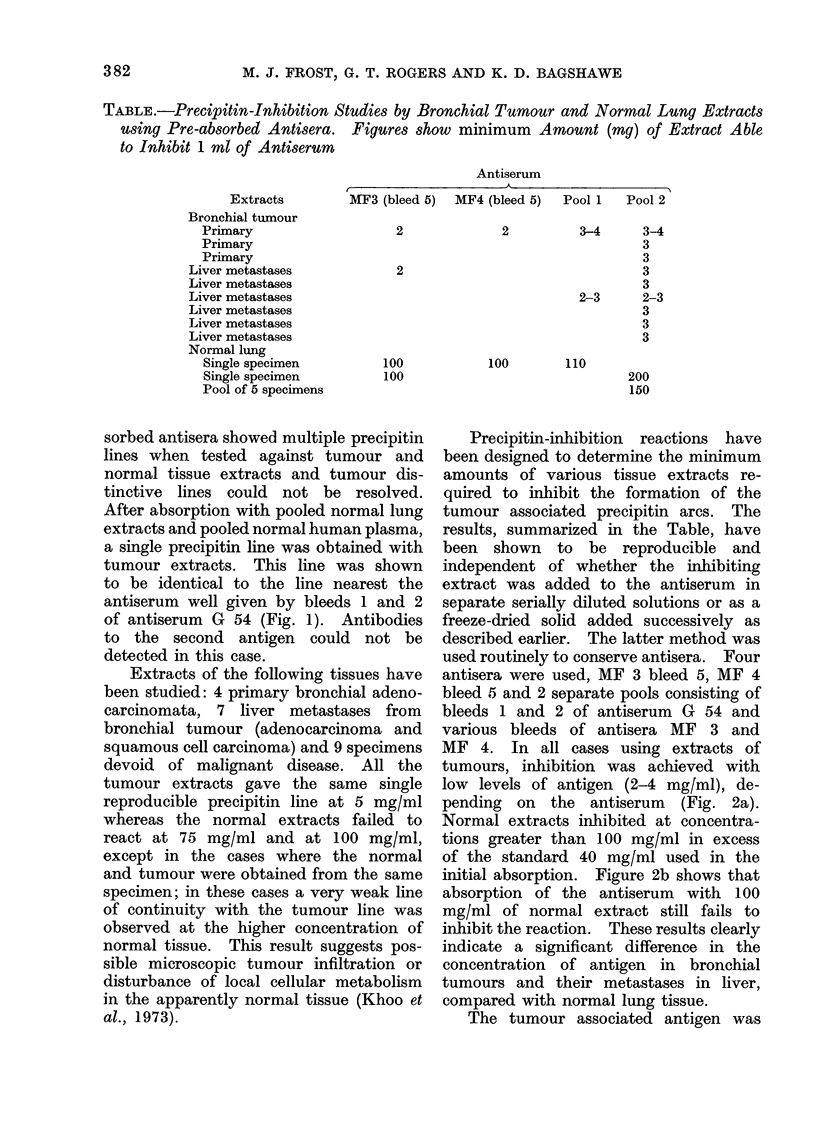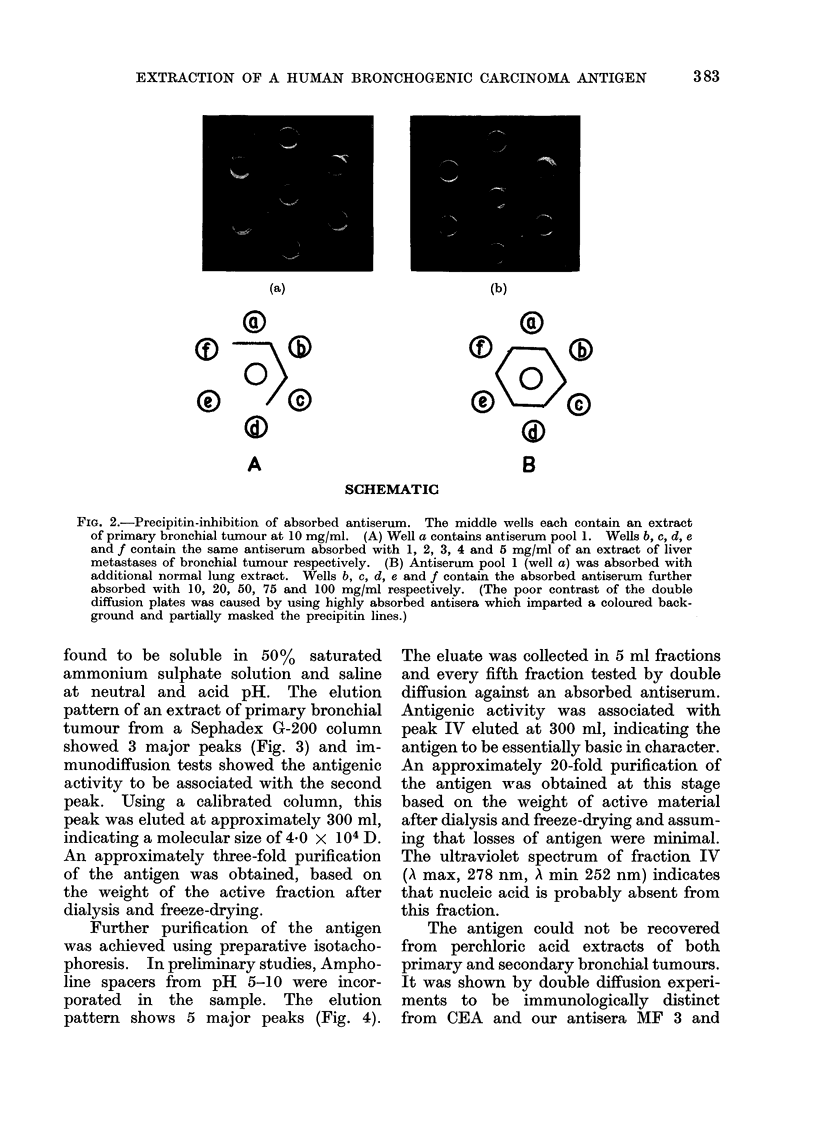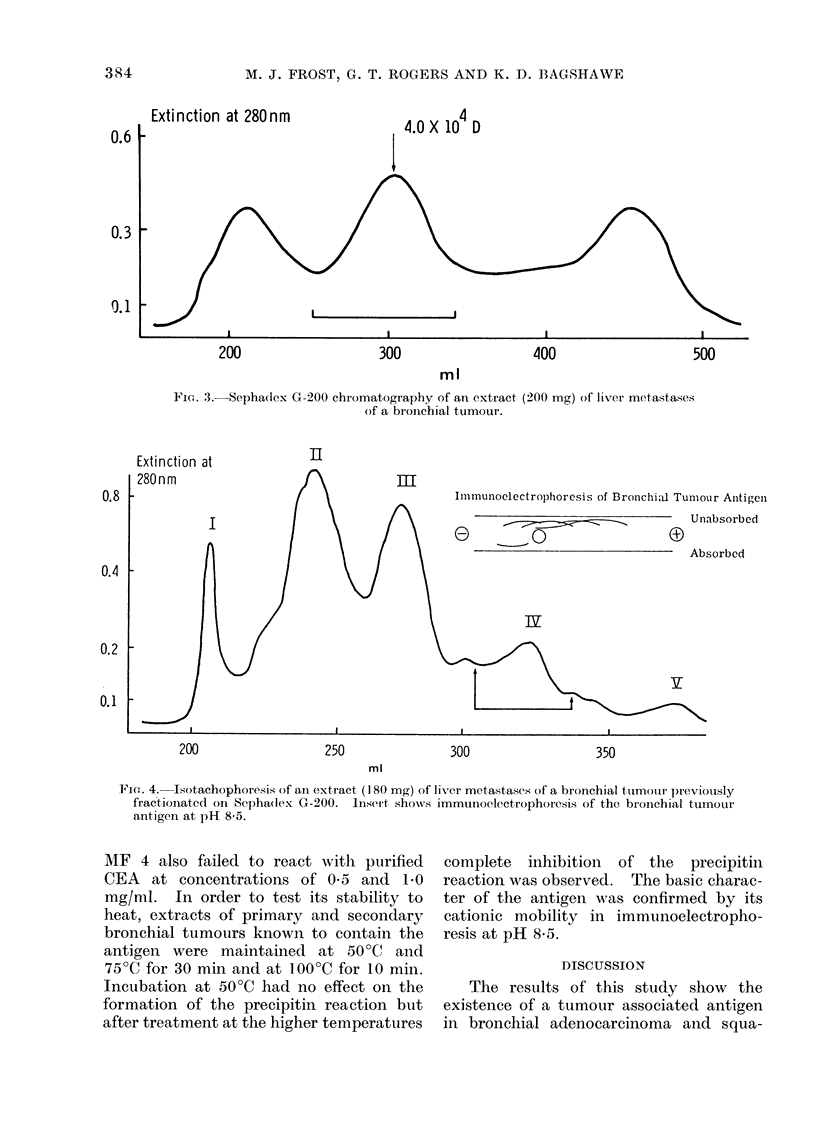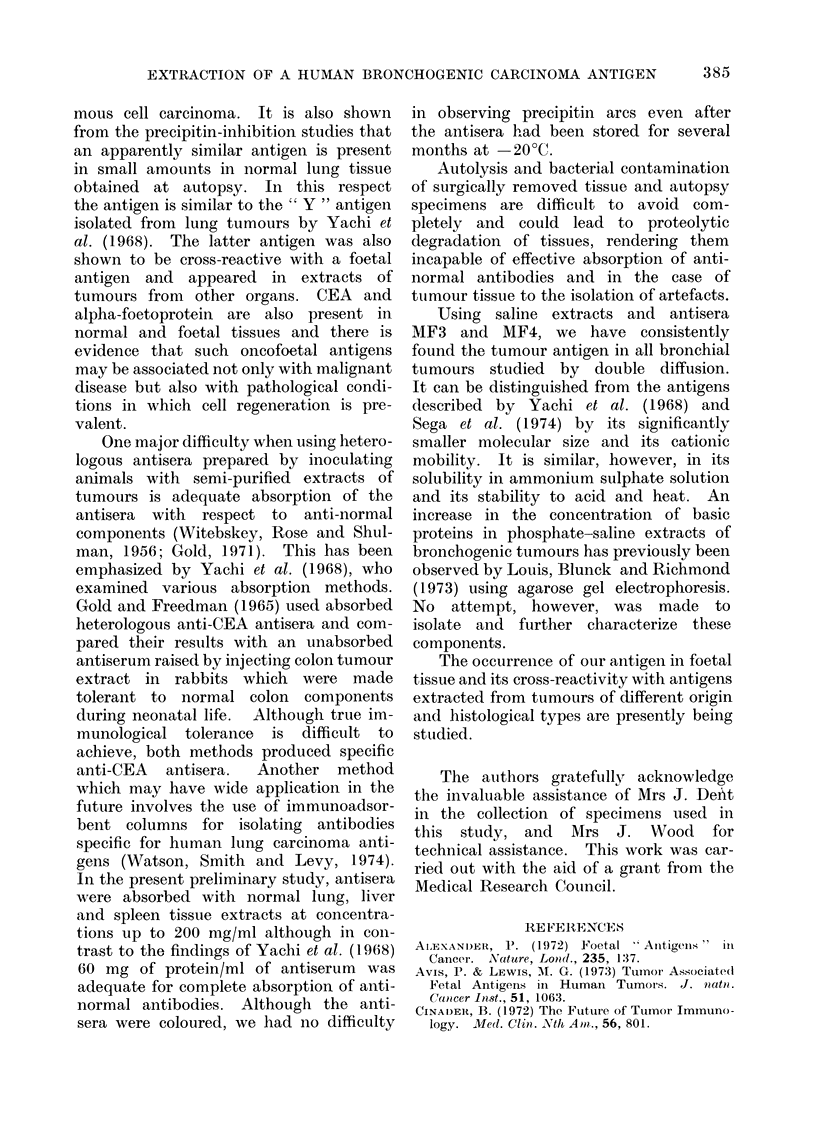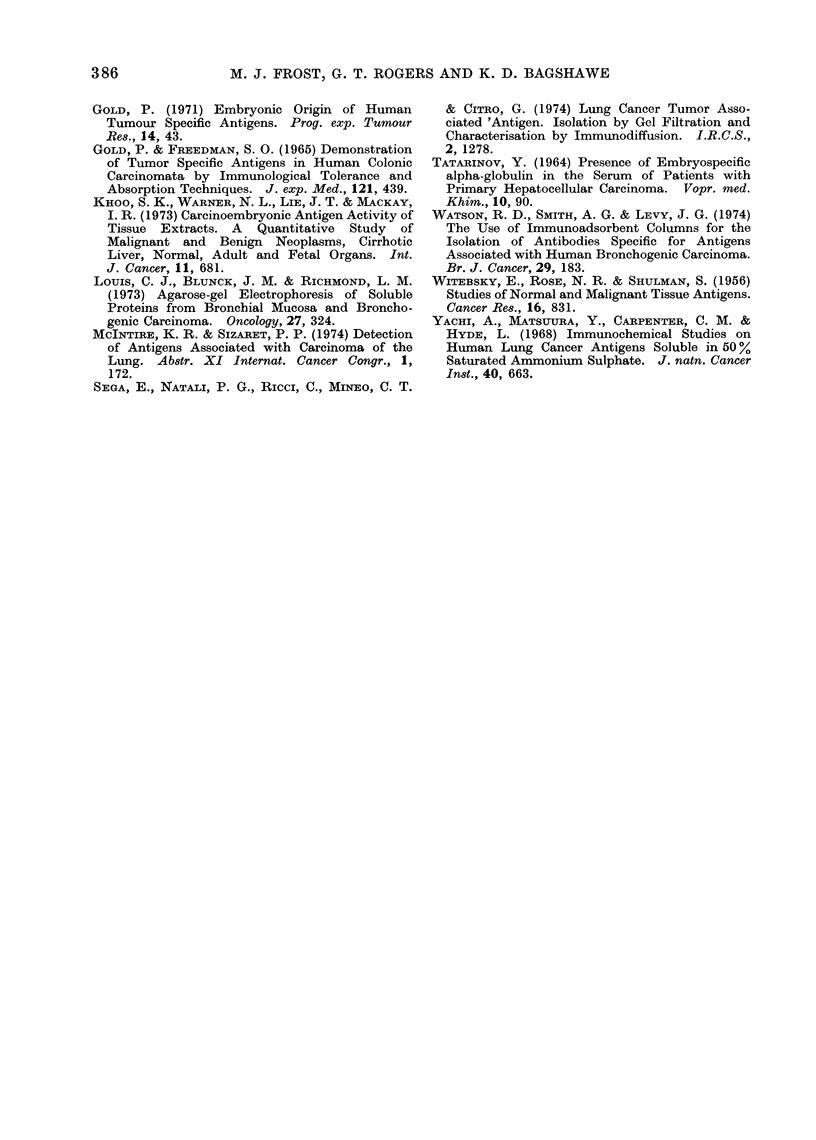# Extraction and preliminary characterization of a human bronchogenic carcinoma antigen.

**DOI:** 10.1038/bjc.1975.77

**Published:** 1975-04

**Authors:** M. J. Frost, G. T. Rogers, K. D. Bagshawe

## Abstract

**Images:**


*Br. J. Cancer* (1975) **31**, 379

### EXTRACTION AND PRELIMINARY CHARACTERIZATION OF A HUMAN BRONCHOGENIC CARCINOMA ANTIGEN

M. J. FROST, G. T. ROGERS AND K. D. BAGSHAWE

*From the Department of Medical Oncology, Charing Cross Hospital, Fulham Palace Road, London W6 8RF*

Received 8 November 1974. Accepted 11 December 1974

**Summary.—Saline extracts of human bronchogenic tumours, soluble in 50% saturated ammonium sulphate and also fractions from Sephadex G-200 chromatography were used to raise antisera in rabbits. After absorbing the antisera with normal tissue extracts, direct Ouchterlony tests were performed against tumour (adenocarcinomata and squamous cell carcinomata) and normal extracts. A precipitin reaction was given with all 11 tumour extracts tested at a concentration of 5 mg/ml whereas all the 9 normal lung control extracts did not react at concentrations up to 100 mg/ml. The possibility that this reaction could be related to histocompatibility differences between individuals is ruled out by the fact that in two cases tumour and normal tissue were obtained from the same patient. These studies and also precipitin-inhibition experiments have confirmed the existence of an antigen associated with bronchial carcinomata and have shown that, although the antigen or a cross-reacting antigen is present in normal lung tissue, the amounts are small in comparison with the amounts extracted from tumour. Antigenic activity was contained in a single absorbance peak when fractionated by Sephadex G-200 chromatography and its elution volume indicated a molecular weight of approximately 4·0 × 10⁴ D. Further purification was achieved using isotachophoresis. Preliminary characterization of the antigen has shown it to be stable at pH 4·5, resistant to heating at 50°C for 30 min, to migrate on immunoelectrophoresis with a cationic mobility at pH 8·5 and to be immunologically distinct from carcinoembryonic antigen.**

IMMUNOCHEMICAL studies have shown the existence of several human tumour associated antigens (Alexander, 1972; Cinader, 1972; Avis and Lewis, 1973). Two of the most widely studied are carcinoembryonic antigen (CEA), originally thought to be specifically associated with colon carcinoma (Gold and Freedman, 1965) and alpha-foetoprotein (Tatarinov, 1964) found in the serum of patients with hepatocellular carcinoma or teratoma. Both of these antigens have been found in foetal tissues. Other studies (Yachi *et al.*, 1968) employing absorbed heterologous antisera have demonstrated by double diffusion at least 2 tumour associated antigens in saline extracts of bronchial carcinomata. These antigens were both soluble in 50% saturated ammonium sulphate but appeared to differ in molecular size. The smaller antigen (7S) in particular was not only related to lung cancer—an identical or partially related antigen was found also in tumours of colon, stomach, pancreas, kidney and liver. It had an electrophoretic mobility in the alpha-beta region (pH 8·6) and is very similar in character to a lung tumour antigen recently reported by Sega *et al.* (1974) although its identity had not been established. Using similar techniques McIntire and Sizaret (1974) have also detected 2 different antigens associated with carcinoma of the lung.

In this paper we present evidence for the existence of a different, more basic, bronchial tumour antigen soluble in 50% saturated ammonium sulphate and report preliminary studies on its purification and characterization.

### MATERIALS AND METHODS

*Specimens.*—Tumour specimens of lung and bronchus, and in 2 cases corresponding normal lung tissue, were obtained surgically. Autopsy specimens obtained within 24 h of death were also used. All tissues were either processed immediately or stored at $-20\,^{\circ}C$ until used.

*Extraction of tissue antigens.*—Neoplastic and normal tissue were extracted using a method similar to that described by Yachi *et al.* (1968). The tissues were cut into small pieces, washed briefly with water to remove blood, minced and homogenized in an equal volume of 0·9% saline for approximately 10 min using a Townson and Mercer top drive macerator. The suspension was then centrifuged at 30,000 *g* for 1 h, the supernatant decanted and the residue resuspended in saline (250 ml/kg of fresh tissue) and recentrifuged. The pooled supernatants were brought to pH 4·5 by adding N HCl dropwise and the precipitate removed by centrifugation at 75,000 *g* for 30 min. The supernatant was concentrated in an Amicon cell using a PM 10 membrane and then fractionated by the dropwise addition of an equal volume of saturated ammonium sulphate solution at 4°C. The stirred suspension was kept at 4°C for 2 h and centrifuged at 30,000 *g* for 1 h. The soluble fraction was then dialysed against running tap water for 2 days, concentrated to about 200 ml (Amicon PM 10), dialysed against distilled water and freeze-dried.

*Antisera.*—A pooled extract of primary well differentiated adenocarcinomata (2 specimens) was used to raise an antiserum (G54) for initial studies. Based on the method of Yachi *et al.* (1968), 15 mg of extract for each rabbit was dissolved in saline (1 ml), sterilized by filtration through a 0·22 $\mu$m Millipore membrane and emulsified in an equal volume of Freund's complete adjuvant. Each rabbit received multiple-site intradermal injections in the flank, a similar booster injection being given approximately 40 days later. Trial bleeds were started 10 days later and the sera absorbed with pooled normal lung extract (40 mg/ml), freeze-dried normal human plasma (20 mg/ml), a saline extract of normal liver and spleen (20 mg/ml) and an extract of lung tissue (20 mg/ml) taken more than 7 cm from the periphery of the tumour used to raise the antiserum. Similar inoculation schedules were employed to raise antisera from antigen containing fractions of extracts of primary bronchial tumour (MF 1 and MF 2) and liver metastases from a squamous cell bronchial tumour (MF 3 and MF 4) which had previously been chromatographed on Sephadex G-200. Each antiserum was absorbed with a pool of normal lung extract (40 mg/ml) and freeze-dried normal human plasma (20 mg/ml) by mixing and leaving at 37°C overnight. The antiserum was centrifuged at 2750 *g* for 20 min and the supernatant filtered through a 0·22 $\mu$m Millipore membrane. Sodium azide was added as a preservative.

*Fractionation of tumour extracts.*—Fractionation of extracts of primary and secondary bronchial tumours was performed with a Sephadex G-200 column (5·3 cm × 89 cm). The column was equilibriated with 0·05 mol/l phosphate buffer at pH 7·2 containing 0·15 mol/l NaCl. Freeze-dried tumour extract (200 mg) was dissolved in this buffer, filtered through a 5 $\mu$m membrane and applied to the column. A flow rate of 20 ml/h was maintained throughout the elution procedure. The effluent samples were monitored by u.v. absorption at 280 nm and tested by Ouchterlony double diffusion using appropriate antisera. Fractions showing a reaction with the absorbed antisera were pooled, dialysed against distilled water and freeze dried. Isotachophoresis was performed using an LKB Uniphore preparative apparatus as described in the operating manual employing 5·5% polyacrylamide gel in a 50 ml column. The leading and terminating buffers were 0·067 mol/l Tris/phosphate pH 6·75 and 0·012 mol/l Tris/0·23 mol/l E-aminocaproic acid pH 8·7 respectively. The sample (180 mg of G-200 fractionated, extract of liver metastases of a bronchial tumour), applied to the column in the terminating buffer (15 ml), incorporated Ampholine spacers (pH 5–10). The flow rate was 20 ml/h and the maximum current applied 15 mA.

*Immunological methods.* — Immunodiffusion experiments were carried out in 1·5%

purified Agar (Oxoid) in 0·9% saline on plates 1·0 mm thick. Wells were 8 mm apart and filled with 10 $\mu$l of antigen solution or antisera. The tumour extracts in saline were generally tested at 5 mg/ml and the normal tissue extracts at a range of concentrations up to 100 mg/ml. Immunoelectrophoresis was carried out in 1·5% Agar gel in 0·05 mol/l barbitone buffer (pH 8·5) using plates 1·0 mm thick. Precipitin-inhibition studies were carried out using absorbed antisera which were treated with successive additions of freeze-dried tumour or normal extracts. The antisera were incubated at 37°C for 2 h and at 4°C overnight after each addition of extract and the centrifuged antiserum tested by direct double diffusion against a primary and secondary bronchial tumour extract (10 mg/ml). Interaction between these extracts and the antiserum absorbed with the standard amounts of normal extracts was previously shown to give a reproducible sharp pattern of a single tumour associated precipitin band.

FIG. 1.—Double diffusion reactions of an extract of primary bronchial tumour (centre well) with various antisera: Wells *a* and *b* G 54; *c* MF4/bleed 7; *d* MF3/bleed 2; and *e* MF3/bleed 7.

### RESULTS

Initial studies were made with antiserum G54. All antiserum bleeds were absorbed as described earlier and tested against various bronchial tumour and normal lung extracts. Bleeds 1 and 2 produced two precipitin arcs (Fig. 1) against the tumour extracts but not against normal extracts at concentrations up to 100 mg/ml. Further absorption of these antisera (up to 200 mg of protein) did not inhibit the formation of these arcs. Subsequent bleeds, however, did not produce precipitin lines after absorption with normal lung extract and a further booster failed to restore anti-tumour activity. Bleeds 1 and 2 from each rabbit were pooled and used for monitoring the Sephadex G-200 fractionation. These initial experiments suggested that possibly two tissue antigens associated with bronchial carcinoma are detectable and although the normal extracts failed to react at the concentrations stated, the possibility of the antigens being present in normal lung tissues at low concentrations was not excluded. These antigens are unlikely to be related to histocompatibility differences between individuals since extracts of corresponding normal and tumour tissue were obtained from the same donors; in addition, absorbed antisera reacted with tumour extracts from many different individuals.

Further antisera were raised using positive fractions obtained from Sephadex G-200 chromatography. Both antigens appeared in a single absorbance peak. Antisera MF 1 and MF 2 produced very weak precipitin lines after absorption and were discarded. Antisera MF 3 and MF 4, however, produced clear reactions after absorption although the lines were partially masked by coloured impurities in the antiserum. Ouchterlony double diffusion reactions using unab-

TABLE.—*Precipitin-Inhibition Studies by Bronchial Tumour and Normal Lung Extracts using Pre-absorbed Antisera. Figures show* minimum *Amount (mg) of Extract Able to Inhibit* 1 *ml of Antiserum*

| | Antiserum | | | |
|---|---|---|---|---|
| Extracts | MF3 (bleed 5) | MF4 (bleed 5) | Pool 1 | Pool 2 |
| Bronchial tumour | | | | |
| Primary | 2 | 2 | 3–4 | 3–4 |
| Primary | | | | 3 |
| Primary | | | | 3 |
| Liver metastases | 2 | | | 3 |
| Liver metastases | | | | 3 |
| Liver metastases | | | 2–3 | 2–3 |
| Liver metastases | | | | 3 |
| Liver metastases | | | | 3 |
| Liver metastases | | | | 3 |
| Normal lung | | | | |
| Single specimen | 100 | 100 | 110 | |
| Single specimen | 100 | | | 200 |
| Pool of 5 specimens | | | | 150 |

sorbed antisera showed multiple precipitin lines when tested against tumour and normal tissue extracts and tumour distinctive lines could not be resolved. After absorption with pooled normal lung extracts and pooled normal human plasma, a single precipitin line was obtained with tumour extracts. This line was shown to be identical to the line nearest the antiserum well given by bleeds 1 and 2 of antiserum G 54 (Fig. 1). Antibodies to the second antigen could not be detected in this case.

Extracts of the following tissues have been studied: 4 primary bronchial adenocarcinomata, 7 liver metastases from bronchial tumour (adenocarcinoma and squamous cell carcinoma) and 9 specimens devoid of malignant disease. All the tumour extracts gave the same single reproducible precipitin line at 5 mg/ml whereas the normal extracts failed to react at 75 mg/ml and at 100 mg/ml, except in the cases where the normal and tumour were obtained from the same specimen; in these cases a very weak line of continuity with the tumour line was observed at the higher concentration of normal tissue. This result suggests possible microscopic tumour infiltration or disturbance of local cellular metabolism in the apparently normal tissue (Khoo *et al.*, 1973).

Precipitin-inhibition reactions have been designed to determine the minimum amounts of various tissue extracts required to inhibit the formation of the tumour associated precipitin arcs. The results, summarized in the Table, have been shown to be reproducible and independent of whether the inhibiting extract was added to the antiserum in separate serially diluted solutions or as a freeze-dried solid added successively as described earlier. The latter method was used routinely to conserve antisera. Four antisera were used, MF 3 bleed 5, MF 4 bleed 5 and 2 separate pools consisting of bleeds 1 and 2 of antiserum G 54 and various bleeds of antisera MF 3 and MF 4. In all cases using extracts of tumours, inhibition was achieved with low levels of antigen (2–4 mg/ml), depending on the antiserum (Fig. 2a). Normal extracts inhibited at concentrations greater than 100 mg/ml in excess of the standard 40 mg/ml used in the initial absorption. Figure 2b shows that absorption of the antiserum with 100 mg/ml of normal extract still fails to inhibit the reaction. These results clearly indicate a significant difference in the concentration of antigen in bronchial tumours and their metastases in liver, compared with normal lung tissue.

The tumour associated antigen was

FIG. 2.—Precipitin-inhibition of absorbed antiserum. The middle wells each contain an extract of primary bronchial tumour at 10 mg/ml. (A) Well *a* contains antiserum pool 1. Wells *b, c, d, e* and *f* contain the same antiserum absorbed with 1, 2, 3, 4 and 5 mg/ml of an extract of liver metastases of bronchial tumour respectively. (B) Antiserum pool 1 (well *a*) was absorbed with additional normal lung extract. Wells *b, c, d, e* and *f* contain the absorbed antiserum further absorbed with 10, 20, 50, 75 and 100 mg/ml respectively. (The poor contrast of the double diffusion plates was caused by using highly absorbed antisera which imparted a coloured background and partially masked the precipitin lines.)

found to be soluble in 50% saturated ammonium sulphate solution and saline at neutral and acid pH. The elution pattern of an extract of primary bronchial tumour from a Sephadex G-200 column showed 3 major peaks (Fig. 3) and immunodiffusion tests showed the antigenic activity to be associated with the second peak. Using a calibrated column, this peak was eluted at approximately 300 ml, indicating a molecular size of $4{\cdot}0 \times 10^4$ D. An approximately three-fold purification of the antigen was obtained, based on the weight of the active fraction after dialysis and freeze-drying.

Further purification of the antigen was achieved using preparative isotachophoresis. In preliminary studies, Ampholine spacers from pH 5–10 were incorporated in the sample. The elution pattern shows 5 major peaks (Fig. 4). The eluate was collected in 5 ml fractions and every fifth fraction tested by double diffusion against an absorbed antiserum. Antigenic activity was associated with peak IV eluted at 300 ml, indicating the antigen to be essentially basic in character. An approximately 20-fold purification of the antigen was obtained at this stage based on the weight of active material after dialysis and freeze-drying and assuming that losses of antigen were minimal. The ultraviolet spectrum of fraction IV ($\lambda$ max, 278 nm, $\lambda$ min 252 nm) indicates that nucleic acid is probably absent from this fraction.

The antigen could not be recovered from perchloric acid extracts of both primary and secondary bronchial tumours. It was shown by double diffusion experiments to be immunologically distinct from CEA and our antisera MF 3 and

FIG. 3.—Sephadex G-200 chromatography of an extract (200 mg) of liver metastases of a bronchial tumour.

FIG. 4.—Isotachophoresis of an extract (180 mg) of liver metastases of a bronchial tumour previously fractionated on Sephadex G-200. Insert shows immunoelectrophoresis of the bronchial tumour antigen at pH 8·5.

MF 4 also failed to react with purified CEA at concentrations of 0·5 and 1·0 mg/ml. In order to test its stability to heat, extracts of primary and secondary bronchial tumours known to contain the antigen were maintained at 50°C and 75°C for 30 min and at 100°C for 10 min. Incubation at 50°C had no effect on the formation of the precipitin reaction but after treatment at the higher temperatures complete inhibition of the precipitin reaction was observed. The basic character of the antigen was confirmed by its cationic mobility in immunoelectrophoresis at pH 8·5.

### DISCUSSION

The results of this study show the existence of a tumour associated antigen in bronchial adenocarcinoma and squamous cell carcinoma. It is also shown from the precipitin-inhibition studies that an apparently similar antigen is present in small amounts in normal lung tissue obtained at autopsy. In this respect the antigen is similar to the " Y " antigen isolated from lung tumours by Yachi *et al.* (1968). The latter antigen was also shown to be cross-reactive with a foetal antigen and appeared in extracts of tumours from other organs. CEA and alpha-foetoprotein are also present in normal and foetal tissues and there is evidence that such oncofoetal antigens may be associated not only with malignant disease but also with pathological conditions in which cell regeneration is prevalent.

One major difficulty when using heterologous antisera prepared by inoculating animals with semi-purified extracts of tumours is adequate absorption of the antisera with respect to anti-normal components (Witebskey, Rose and Shulman, 1956; Gold, 1971). This has been emphasized by Yachi *et al.* (1968), who examined various absorption methods. Gold and Freedman (1965) used absorbed heterologous anti-CEA antisera and compared their results with an unabsorbed antiserum raised by injecting colon tumour extract in rabbits which were made tolerant to normal colon components during neonatal life. Although true immunological tolerance is difficult to achieve, both methods produced specific anti-CEA antisera. Another method which may have wide application in the future involves the use of immunoadsorbent columns for isolating antibodies specific for human lung carcinoma antigens (Watson, Smith and Levy, 1974). In the present preliminary study, antisera were absorbed with normal lung, liver and spleen tissue extracts at concentrations up to 200 mg/ml although in contrast to the findings of Yachi *et al.* (1968) 60 mg of protein/ml of antiserum was adequate for complete absorption of anti-normal antibodies. Although the antisera were coloured, we had no difficulty in observing precipitin arcs even after the antisera had been stored for several months at −20°C.

Autolysis and bacterial contamination of surgically removed tissue and autopsy specimens are difficult to avoid completely and could lead to proteolytic degradation of tissues, rendering them incapable of effective absorption of anti-normal antibodies and in the case of tumour tissue to the isolation of artefacts.

Using saline extracts and antisera MF3 and MF4, we have consistently found the tumour antigen in all bronchial tumours studied by double diffusion. It can be distinguished from the antigens described by Yachi *et al.* (1968) and Sega *et al.* (1974) by its significantly smaller molecular size and its cationic mobility. It is similar, however, in its solubility in ammonium sulphate solution and its stability to acid and heat. An increase in the concentration of basic proteins in phosphate–saline extracts of bronchogenic tumours has previously been observed by Louis, Blunck and Richmond (1973) using agarose gel electrophoresis. No attempt, however, was made to isolate and further characterize these components.

The occurrence of our antigen in foetal tissue and its cross-reactivity with antigens extracted from tumours of different origin and histological types are presently being studied.

The authors gratefully acknowledge the invaluable assistance of Mrs J. Dent in the collection of specimens used in this study, and Mrs J. Wood for technical assistance. This work was carried out with the aid of a grant from the Medical Research Council.

REFERENCES

ALEXANDER, P. (1972) Foetal " Antigens " in Cancer. *Nature, Lond.*, **235**, 137.

AVIS, P. & LEWIS, M. G. (1973) Tumor Associated Fetal Antigens in Human Tumors. *J. natn. Cancer Inst.*, **51**, 1063.

CINADER, B. (1972) The Future of Tumor Immunology. *Med. Clin. Nth Am.*, **56**, 801.

GOLD, P. (1971) Embryonic Origin of Human Tumour Specific Antigens. *Prog. exp. Tumour Res.*, **14**, 43.

GOLD, P. & FREEDMAN, S. O. (1965) Demonstration of Tumor Specific Antigens in Human Colonic Carcinomata by Immunological Tolerance and Absorption Techniques. *J. exp. Med.*, **121**, 439.

KHOO, S. K., WARNER, N. L., LIE, J. T. & MACKAY, I. R. (1973) Carcinoembryonic Antigen Activity of Tissue Extracts. A Quantitative Study of Malignant and Benign Neoplasms, Cirrhotic Liver, Normal, Adult and Fetal Organs. *Int. J. Cancer*, **11**, 681.

LOUIS, C. J., BLUNCK, J. M. & RICHMOND, L. M. (1973) Agarose-gel Electrophoresis of Soluble Proteins from Bronchial Mucosa and Bronchogenic Carcinoma. *Oncology*, **27**, 324.

MCINTIRE, K. R. & SIZARET, P. P. (1974) Detection of Antigens Associated with Carcinoma of the Lung. *Abstr. XI Internat. Cancer Congr.*, **1**, 172.

SEGA, E., NATALI, P. G., RICCI, C., MINEO, C. T. & CITRO, G. (1974) Lung Cancer Tumor Associated Antigen. Isolation by Gel Filtration and Characterisation by Immunodiffusion. *I.R.C.S.*, **2**, 1278.

TATARINOV, Y. (1964) Presence of Embryospecific alpha-globulin in the Serum of Patients with Primary Hepatocellular Carcinoma. *Vopr. med. Khim.*, **10**, 90.

WATSON, R. D., SMITH, A. G. & LEVY, J. G. (1974) The Use of Immunoadsorbent Columns for the Isolation of Antibodies Specific for Antigens Associated with Human Bronchogenic Carcinoma. *Br. J. Cancer*, **29**, 183.

WITEBSKY, E., ROSE, N. R. & SHULMAN, S. (1956) Studies of Normal and Malignant Tissue Antigens. *Cancer Res.*, **16**, 831.

YACHI, A., MATSUURA, Y., CARPENTER, C. M. & HYDE, L. (1968) Immunochemical Studies on Human Lung Cancer Antigens Soluble in 50% Saturated Ammonium Sulphate. *J. natn. Cancer Inst.*, **40**, 663.